# Editorial: Strategies to overcome tumor evasion and resistance to immunotherapies by targeting immune suppressor cells

**DOI:** 10.3389/fonc.2023.1240926

**Published:** 2023-07-07

**Authors:** Yu Saida, Satoshi Watanabe, Shohei Koyama, Yosuke Togashi, Toshiaki Kikuchi

**Affiliations:** ^1^Department of Respiratory Medicine and Infectious Diseases, Niigata University Graduate School of Medical and Dental Sciences, Niigata, Japan; ^2^Division of Cancer Immunology, Research Institute/Exploratory Oncology Research and Clinical Trial Center, National Cancer Center, Tokyo, Japan; ^3^Department of Tumor Microenvironment, Okayama University, Graduate School of Medicine Dentistry and Pharmaceutical Sciences, Okayama, Japan

**Keywords:** tumor evasion, immunotherapy, immunosuppressive cells, tumor microenvironment, antitumor immune response

Cancer immunotherapy has advanced during the past few decades. Particularly, immune checkpoint inhibitors (ICIs) targeting PD-1/PD-L1 and CTLA-4 have now become standard therapy for several cancers. A key feature of immunotherapy is a durable tumor response, which results in a plateau at the tail of the survival curve. Despite their potential, significant responses to ICIs are limited to a fraction of patients. Several mechanisms of resistance to ICIs have been proposed: the lack of neo-antigens, impaired presentation of tumor antigens, metabolic/inflammatory mediators, immune suppressor cells, inhibitory signals *via* alternate immune checkpoints, and T cell exhaustion. Regulatory T cells, myeloid-derived suppressor cells, tumor-associated macrophages, and tumor-associated neutrophils are major immune suppressor cells that inhibit antitumor immune responses at the tumor microenvironment (TME). Also, cancer-associated fibroblasts are complex barriers for immune cells. Modulating TME is a potential strategy for enhancing the efficacy of immunotherapy. The objective of this Research Topic was to improve our understanding of the contribution of TME to immunotherapy resistance and obtain indicators for the development of novel immunotherapies.

Zhao and Liu focused on P4HA1, a key enzyme in collagen synthesis, as a potential prognostic marker and its impact on TME. Bioinformatics analysis of pan-cancer cells revealed that P4HA1 expression correlated with poor survival and infiltration levels of immunosuppressive cells, such as tumor-associated macrophages, cancer-associated fibroblasts, and regulatory T cells, whereas it negatively correlated with effector cells, such as CD8^+^ T and natural killer cells. Hence, the extracellular matrix (ECM) is a significant physical barrier within the tumor that needs to be overcome to maximize immunotherapy efficacy. P4HA1 may be a potential target to improve the TME for cancer treatment because P4HA1-mediated high collagen deposition is critical for tumor progression.

Chen et al. reviewed other ECM factors, including neutrophil extracellular traps, particularly in digestive cancers. Discoidin domain receptor tyrosine kinase 1 is a specific receptor for collagen and is a central mediator of tumorigenic collagen signal transduction. The activation of this kinase by collagen promotes tumor-associated neutrophil invasion and neutrophil extracellular trap formation, causing cancer cell invasion and metastasis in pancreatic ductal adenocarcinoma (1). Therefore, discoidin domain receptor tyrosine kinase 1 may be a potential target for altering neutrophil extracellular trap expression.

Bruni et al. reported hyaluronan (HA), which is another component of the ECM. High HA expression correlates with a poor prognosis in patients with pancreatic ductal adenocarcinoma. Recently, the PEGylated human hyaluronidase (PEGPH20) was reported to be potentially useful for the radiosensitization of pancreatic ductal adenocarcinoma (2). Additionally, PEGPH20 increases the uptake of PD-L1 antibodies in HA-accumulating breast cancer, suggesting that PEGPH20 can sensitize HA-accumulating tumors to anti-PD-L1 therapy (3); however, the phase III trial of PEGPH20 in combination with nab-paclitaxel/gemcitabine failed to influence overall survival (4). Overcoming the physical barriers of the ECM remains a potential strategy to enhance cancer immunotherapy.

Luo et al. performed a meta-analysis of data from TCGA and CGGA for patients with low-grade glioma. Increased levels of naïve CD4^+^ T cells activated mast cells and monocytes and were correlated with a better prognosis, whereas higher abundances of resting memory CD4^+^ T cells and M1 macrophages were correlated with poor overall survival. Through their analysis, the authors constructed a risk signature based on immune cell infiltration to predict the prognosis of patients with low-grade glioma. Thus, in understanding the immune status of cancer, both host and tumor factors must be considered. The complexity of these factors makes the prediction of immunotherapy efficacy using a single biomarker difficult. In addition to the immune status of the cancer, gene mutations should be considered to predict immunotherapy efficacy. Among patients with non-small-cell lung cancer, PIK3CA, EGFR, or STK11 mutations did not respond to ICIs, whereas KRAS, TP53 mutants, and mesenchymal-to-epithelial transition factor gene exon 14 skipping mutations responded well to ICIs (5). Further research is required to explore potential biomarkers of driver genes to predict ICI efficacy.

Cao et al. reviewed a strategy for targeting myeloid-derived immune suppressor cells in the TME. In this review, they focused on myeloid-derived suppressor cell-directed therapeutics, such as CSF-R1 inhibitors or multi-function TKIs, as well as propranolol, a non-selective blocker, for their potential ability to reprogram immunosuppressive cells within the TME. Recently, the growth inhibitory activity of 4,518 existing non-oncological drugs was tested across 578 human cancer cell lines, resulting in the identification of 49 drugs that selectively killed cancer cells and had anticancer activity predictable by biomarkers (6). Thus, “drug repositioning” appears to be an important strategy, as drugs modulating TME do not necessarily have a direct antitumor effect; however, there are several issues with drug repositioning, such as the expansion of patents to include other diseases and low drug prices due to the use of existing product indications.

Katakai reviewed the role of B lymphocytes in antitumor immunity, immune tolerance in solid tumors, and immune checkpoint inhibitors. The author suggested that antitumor and tumor-suppressive B cell functions were linked to the degree of the antitumor immune response in mid- and late-stage tumor-draining lymph nodes. This perspective is closely related to the selection of perioperative cancer treatments. First, a careful decision is required for lymph node dissection regarding the preservation of the antitumor immune effects. Second, the timing of perioperative immunotherapy should be considered to maximize its efficacy. For example, in addition to adjuvant PD-L1 inhibitors, a neoadjuvant PD-1 inhibitor in combination with chemotherapy was approved for non-small-cell lung cancer with a good pathological and complete response and event-free survival in clinical trials (7, 8). Therefore, optimal operative procedures, including reduced resection, should be considered.

In conclusion, there are many candidates for enhancing immunotherapy other than therapeutics that directly target immunosuppressive cells. Anti-angiogenic therapy is a typical approach for TME modulation. In addition, targeting the gut microbiome in terms of cancer immunity has recently garnered interest. Thus, a multidisciplinary strategy is required to overcome barriers to immunotherapy. Further investigations into novel therapies and drug repositioning are required.

## Author contributions

All authors contributed to the Research Topic editorials and performed the literature searches. YS and SW drafted the manuscript. SW revised the manuscript critically. All the authors have read and approved the final version of the manuscript.
